# Gaussian-Fitting-Enabled High-Accuracy pH Detection for Light-Addressable Potentiometric Sensor

**DOI:** 10.3390/s26051465

**Published:** 2026-02-26

**Authors:** Jie Tan, Zigeng Huang, Bin Sun, Xin Cao, Zijie Tang, Guomao Yan, Jiangze Ren, Shibin Liu, Yinghao Chen, Guifang Li, Xueliang Li, Dong Chen

**Affiliations:** 1 School of Electrical Engineering and Electronic Information, Xihua University, Chengdu 610097, China; zghsczt66@163.com (Z.H.); 13721360102@163.com (B.S.); cuitcx@163.com (X.C.); 18180318380@163.com (Z.T.); yanguomao11@163.com (G.Y.); m18398422628@163.com (J.R.); 2 School of Electronics and Information, Northwestern Polytechnical University, Xi’an 710072, China; liushibin@nwpu.edu.cn (S.L.); 18829589303@163.com (Y.C.); gfli@nwpu.edu.cn (G.L.); 3 School of Mechanical and Electrical Engineering, Zhoukou Normal University, Zhoukou 466001, China; zhf870721@zknu.edu.cn; 4 School of Electronics Engineering, Xi’an University of Posts and Telecommunications, Xi’an 710121, China; dchen402@126.com

**Keywords:** light-addressable potentiometric sensor, pH detection, depletion region, Gaussian function, high detection performance

## Abstract

A light-addressable potentiometric sensor (LAPS) is a low-cost and versatile semiconductor field-effect pH sensor. In practical application, typical pH detection based on LAPS usually adopts the method of normalizing the voltage–photocurrent (V-I) characteristic to solve the working point. However, this method not only needs to obtain the data of the whole V-I characteristic, which leads to slow and time-consuming measurement, but the selection of the working point is also greatly influenced by the shape and noise of the V-I characteristic. In view of this, a new pH measurement method is proposed in this paper, which reduces the impact of noise fluctuations by fitting a Gaussian function to the local depletion region of the V-I characteristic and is almost unaffected by some measurement points distortions of the V-I characteristic, and the measurement results are directly obtained from robust morphological parameters of the fitted function. The experimental results show that the new measurement method can not only obtain pH detection with high sensitivity, high linearity and strong specificity but also further improve the detection speed by shortening the range of the bias voltage, reducing the number of measurement points, and increasing the step value of the bias voltage. At the same time, the measurement method has strong anti-interference ability when the light source fluctuates and is applicable to a variety of waveform excitation scenarios. In practical application, this measurement method has low errors in the pH detection of sewage samples. The measurement method expands the measurement mode of LAPS and provides a new idea for high-precision, rapid pH detection and other biochemical species detection marked by pH.

## 1. Introduction

A light-addressable potentiometric sensor (LAPS) is a kind of potentiometric chemical/biological sensor with an electrolyte–insulator–semiconductor structure based on the semiconductor field effect principle [1,2], and it has the advantages of simple structure, high sensitivity, low cost, and compatibility with the traditional integrated circuit process. Therefore, LAPSs have been widely used in biochemical analysis [3,4], medical diagnosis [5,6], food safety [7,8], and environmental monitoring [9,10,11], etc.

In many biochemical reactions, the participation of protons or hydroxide ions is crucial, so pH is often regarded as one of the important indicators in biochemical reactions [12,13,14,15], which directly or indirectly reflects the process, scale, rate and products of biochemical reactions. Depending on the different pH of the electrolyte, the interfacial potential caused by the interaction of the sensing film on the surface of LAPSs with protons or hydroxide ions in the electrolyte changes accordingly. To detect this change, a suitable bias voltage must be selected so that a depletion layer for charge separation is created near the interface between the semiconductor and the insulator layer. When the semiconductor is illuminated with a beam of AC-modulated light whose incident photon energy is greater than the bandgap of the semiconductor, the semiconductor intrinsically absorbs photons and produces photogenerated electron–hole pairs, which repeatedly charge–discharge the depletion layer. In addition, information about the thickness of the depletion layer can be obtained by detecting the amplitude and phase of the AC photocurrent flowing through the LAPS, thus obtaining a function of the pH and interfacial potential [16,17]. To reduce the influence of the electrolyte conductivity on measurement results of LAPSs, the traditional measurement mode usually needs to normalize the photocurrent signal under voltage scanning conditions within a predetermined bias range, and the normalized voltage–photocurrent (V-I) characteristics should be plotted. The V-I characteristics linearly shift left or right along the bias voltage axis when the pH changes, so the point where the normalized photocurrent is 0.5 is usually used as the working point to fit the displacement of bias voltage with pH to get the sensitivity and linearity of pH detection, which is the most classical and accurate method at present [18]. However, this method needs to acquire the whole V-I characteristics for each measurement, which is time-consuming due to the large amount of data. During actual measurement, normalizing V-I characteristics is easily affected by measurement points distortions, instrument measurement noise and environmental noise. Therefore, using a point where the normalized photocurrent is 0.5 as the working point reduces pH detection accuracy. To shorten the measurement time, researchers developed the constant-voltage method, which obtains the pH change information by recording the photocurrent change with a constant voltage [19]. However, this method is limited by the size of the depletion region, resulting in a low pH detection accuracy. To avoid the influence of distortions and noise of the V-I characteristics on measurement results, Yoshinobu proposed a constant-current measurement method, in which the change in bias voltage is recorded by setting the output photocurrent to a constant value to characterize the temporal change in pH. To accurately determine the position of the working point, the constant-current method requires the acquisition of the entire V-I characteristics [20]. To shorten the measurement time and improve the measurement accuracy at the same time, Miyamoto proposed a potential-tracking method, which reconstructs the V-I characteristics and calculates the horizontal displacement through a logistic model. This method greatly reduces the measurement time and can measure larger pH changes [21].

In this paper, a new pH measurement method based on LAPS is proposed, which uses the local depletion region driven by periodic inverted-triangular bias voltage to generate photocurrent waveforms close to Gaussian distribution and converts pH-dependent lateral displacement into robust morphological parameters extracted from a fitting function. Since the positive travel and negative travel scans are completed within the same cycle, the information utilization rate of the V-I characteristic doubles. The results show that compared with the traditional measurement method, the measurement method proposed in this paper does not require full curve normalization or the selection of a single working point but only needs to obtain and fit a small amount of photocurrent data in the local depletion region to obtain the pH measurement results with high sensitivity, high linearity, strong specificity, strong anti-interference and universal light excitation, thereby improving the anti-interference ability against point-like distortions and light fluctuations.

## 2. Experimental Work and Theory

### 2.1. Fabrication of LAPS Chip

The typical LAPS structure used in this paper is a stacked structure composed of Si, SiO_2_, Si_3_N_4_, Cr and Au [22]. The semiconductor is n-type single-crystal silicon with a crystal orientation of <100>, resistivity of 1~10 Ω·cm and thickness of 100 μm. The insulator layer is 50 nm thick SiO_2_, the sensing film is 50 nm thick Si_3_N_4_, and the working electrodes are Cr/Au. The fabrication process is shown in Process S1 and Figure S1.

### 2.2. Characterization of LAPS Chip

The fabricated LAPS chip was micro-characterized by scanning electron microscopy (SEM) to determine its morphology, and energy dispersive X-ray spectroscopy (EDS) combined with SEM was used to scan the cross-section of the LAPS to determine its layered structure and elemental composition. Figure S2 shows the microscopic morphology of the LAPS chip at 2000× and 100,000× magnification and the corresponding EDS surface scan images of the cross-section. According to the scan images, the main component of the LAPS chip is silicon. The presence of nitrogen and oxygen elements on the silicon indicates the existence of SiO_2_ and Si_3_N_4_ films. In addition, the scattered distribution of chromium and gold elements beneath the silicon indicates the presence of metal electrodes. The results show that the LAPS chip has a layered structure of Si_3_N_4_/ SiO_2_/Si/Cr/Au.

### 2.3. The Construction of the Measurement System

**Figure 1:**
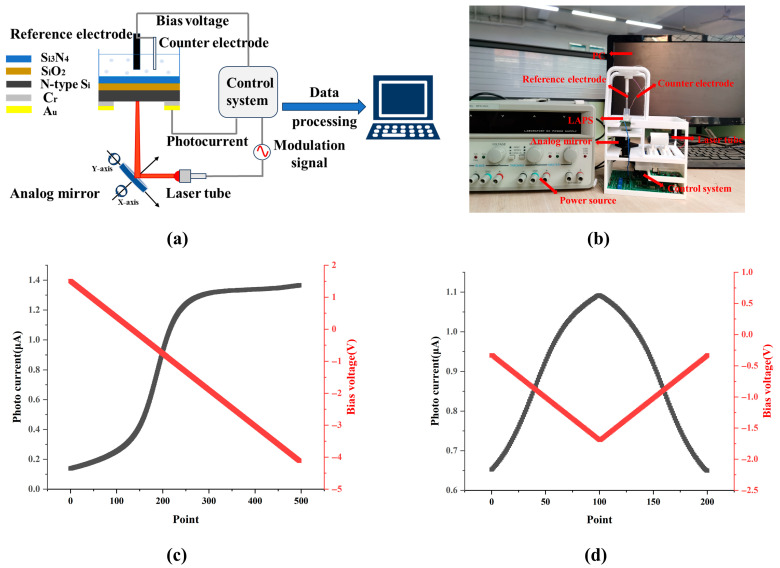
Measurement system and bias voltage setting. (**a**) Schematic diagram of measurement system. (**b**) Physical diagram of measurement system. Applied bias voltage curves and corresponding photocurrent curves using the VICM method (**c**) and the GFFM method (**d**).

The package of the LAPS chip is shown in Figure S3. The fabricated LAPS chip is encapsulated on a printed circuit board (PCB) by conductive silver gel, the size of the bottom window of PCB is set to 1 cm × 1 cm for illumination, and the glass tube is glued to the surface of the sensing film of the LAPS chip for containing the electrolyte. Figure 1a shows a schematic diagram of the LAPS measurement system, which mainly includes the encapsulated LAPS chip, DC power supply, analog mirror, control system, reference electrode, counter electrode, laser tube, and computer. The control system is a self-designed embedded system, whose main functions include providing bias voltage for the LAPS through the reference electrode and counter electrode, acquiring the output photocurrent of the LAPS, providing light source excitation signals for the laser tube, and pre-processing the photocurrent signal. The laser tube is a semiconductor laser with a wavelength of 638 nm, and its light power is regulated by the AC current flowing through the laser [23]. The analog mirror is responsible for changing the direction of the beam so that it is incident perpendicular to the substrate surface of the LAPS. The computer is responsible for processing the measurement results from the control system and visualizing them. A physical diagram of the measurement system is shown in Figure 1b.

### 2.4. Setting the Bias Voltage

The bias voltage setting and the photocurrent curve are shown in Figure 1c when the traditional V-I characteristic measurement (VICM) method is used. The bias voltage ranges from −4.124 V to 1.53 V, the number of measurement points is 500, the change shape is stepped, and the step value is 11.3 mV. The excitation signal of the light source adopts a sinusoidal waveform, and the modulation frequency is set to 10 kHz. To ensure that the LAPS operates in the depletion region, the bias voltage setting and the photocurrent curve are shown in Figure 1d when the Gaussian function fitting measurement (GFFM) method is adopted. The range of the bias voltage is set from −1.69 V to −0.334 V, 120 measurement points are included in the bias voltage range and the change shape is a periodic inverted triangle, so the step value is 11.3 mV. In addition, the excitation signal of the light source also adopts a sinusoidal waveform with a modulation frequency of 10 kHz.

### 2.5. Theoretical Analysis of the Gaussian Function Fitting Measurement Method

**Figure 2:**
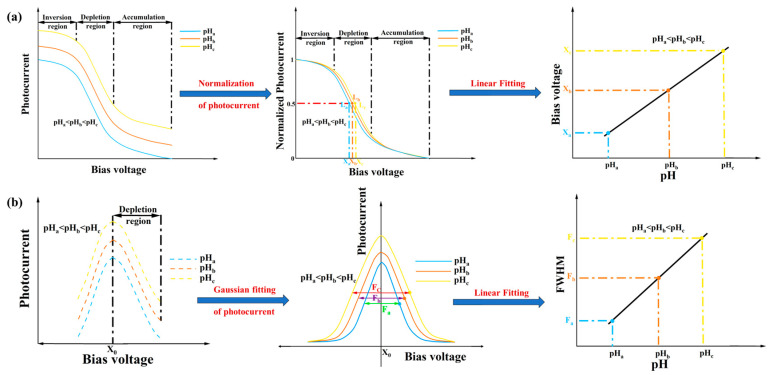
The schematic diagram of the two measurement methods. (**a**) The traditional VICM method; (**b**) the GFFM method.

The traditional VICM method is shown schematically in Figure 2a. This method requires the bias voltage scan range of the V-I characteristics containing the inversion region, depletion region, and accumulation region to be determined in advance, and then the photocurrent signal is acquired by applying a corresponding scan voltage. After that, the V-I characteristics under each pH condition are normalized, and normalized V-I characteristics shift left or right along the voltage axis as the pH changes. According to the Nernst equation, the interfacial potential ϕ and pH can be expressed as follows:

(1) $$\phi =E_{0}+k\cdot \mathrm{pH}$$

where $E_{0}$ is the potential constant and k is a constant parameter. Therefore, the dependence of pH and ϕ is linear. However, the actual field effect inside the LAPS is determined by both the bias voltage and the interface potential, and the sensitivity only needs to calculate the lateral shift in bias voltage versus pH when the normalized photocurrent is constant. A point with a normalized photocurrent of 0.5 at each pH condition is set as the working point (that is, L_a_, L_b_ and L_c_ in Figure 2a are the working points of pH_a_, pH_b_ and pH_c_, respectively) of the LAPS at that pH condition. The characteristics of pH measurement such as sensitivity and linearity can be obtained by plotting a scatter plot of the horizontal coordinates of the working point and pH and fitting the data linearly.

By observing Figure 1d, we find that the output photocurrent curve of the LAPS is similar to the Gaussian function curve under the application of a one-cycle inverted-triangular-shaped bias voltage [24,25], so we can use the Gaussian function to fit the output photocurrent. The Gaussian function model is shown in Equation (2):

(2) $$y=M\cdot e^{-{\left(x-x_{0}\right)}^{2}/c^{2}}$$

where y is the output, M is the height of the Gaussian function curve, $x_{0}$ is the position of the horizontal coordinate corresponding to the maximum value of the function, and c is 1.414 times the standard deviation of the function. The full width at half maximum (FWHM) serves as a fundamental metric for quantifying the broadening characteristics of Gaussian distributions, with its magnitude directly determining the spread of the function. The FWHM is rigorously defined as

(3) $$\mathrm{FWHM}=2\sqrt{\ln2}\cdot c$$

Consequently, variations in FWHM provide quantitative insight into morphological changes in the Gaussian curve. The schematic diagram of the GFFM method is shown in Figure 2b. By fitting the Gaussian function to the photocurrent obtained from the LAPS with a single-cycle inverted-triangular bias voltage applied, it can be found that the spread degree of the fitted curve becomes greater as pH increases, and morphological changes in the curve shows a linear relationship with pH. Therefore, there is no need to consider the differences in the positions of the Gaussian curves, and only the scatter plot of FWHM (that is, F_a_, F_b_ and F_c_ in Figure 2b are FWHM of pH_a_, pH_b_ and pH_c_, respectively) and pH needs to be plotted, and they need to be fit linearly to obtain the sensitivity and linearity characteristics of the GFFM method for measuring pH. When the GFFM method is used in actual measurement, the overall displacements of the depletion region of the V-I characteristics with pH changes are considered, which compensates for measurement errors in calculating the displacement of individual working points due to some measurement point distortions and noise fluctuations and can directly respond to the pH measurement results while reducing the number of data acquisitions.

## 3. Result and Discussion

### 3.1. V-I Characteristics Measurement Method

**Figure 3:**
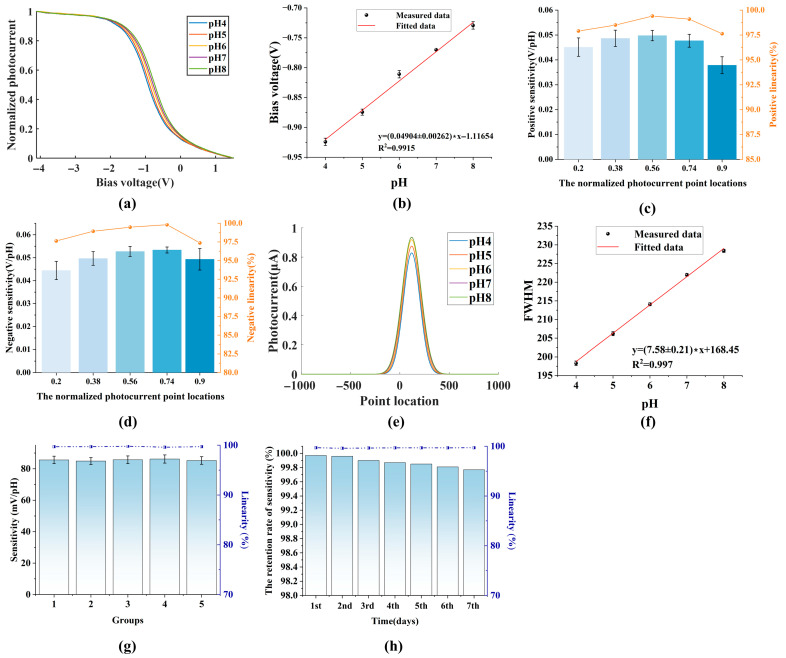
The measurement curves of the traditional VICM method and the GFFM method. (**a**) Normalized V-I characteristics at different pH using the traditional VICM method. (**b**) Sensitivity and linearity of the LAPS obtained by the traditional VICM method. (**c**) The positive sensitivities and the positive linearities of the LAPS when the normalized photocurrent point locations are 0.9, 0.74, 0.56, 0.38, and 0.2, respectively (positive travel: the bias voltage sweeps from small to large). (**d**) The negative sensitivities and the negative linearities of LAPS when the normalized photocurrent point locations are 0.9, 0.74, 0.56, 0.38, and 0.2, respectively (negative travel: the bias voltage sweeps from large to small). (**e**) Gaussian function fitting curves under different pH conditions. (**f**) The sensitivity and linearity of the LAPS obtained by the GFFM method. (**g**) The comparison of sensitivities and linearities of five independent LAPSs using the GFFM method. (**h**) The time changes in the sensitivities and linearities of an independent LAPS using the GFFM method.

When the LAPS is measured using the traditional VICM method, the light source setting and bias voltage setting are elaborated in Section 2.4, and the measured results are shown in Figure 3a. From Figure 3a, when the applied bias voltage ranges from negative to positive, the photocurrents of the V-I characteristics vary from high to low during the transition from the inversion region to the accumulation region. In addition, with the increase in the pH of the measured electrolyte, more hydroxide ions are adsorbed onto the surface of the silicon nitride layer, inducing additional negative potentials and causing the whole V-I characteristics to shift in the positive direction along the voltage axis. Figure 3b shows the sensitivity and linearity measured using the VICM method, and ten measurements are conducted under each pH condition. The sensitivity and the linearity are 49.04 mV/pH and 99.15%, respectively. At the same time, we calculate the limit of detection (LOD) of the VICM method combining the sensitivities and standard deviation based on the LOD definition of IUPAC, and the value is 0.32 pH. Further, we calculate the horizontal displacement in the V-I characteristics by selecting different normalized photocurrent point locations, and the measured results for positive travel (the bias voltage sweeps from small to large) and negative travel (the bias voltage sweeps from large to small) are shown in Figure 3c,d. When the normalized photocurrent point locations are 0.9, 0.74, 0.56, 0.38, and 0.2, the positive sensitivities of the LAPS are 37.86 mV/pH, 47.7 mV/pH, 49.79 mV/pH, 48.66 mV/pH, and 45.14 mV/pH, respectively. The corresponding negative sensitivities are 49.33 mV/pH, 53.37 mV/pH, 52.73 mV/pH, 49.65 mV/pH, and 44.44 mV/pH, respectively. When normalized photocurrent point locations of 0.9, 0.74, 0.56, 0.38, and 0.2 are chosen, the sensitivities of the LAPS are different, with greater sensitivities at the middle position and less sensitivities away from the middle position. This indicates that the displacement calculation of the depletion region with pH change inevitably contains measurement errors due to instrument noise, environmental noise, etc. In addition, the positive linearities are 97.63%, 99.1%, 99.4%, 98.5%, and 97.9%, respectively, and the negative linearities are 97.36%, 99.81%, 99.48%, 98.93%, and 97.63%, respectively. In addition, the linearities at the middle of the depletion region are similarly superior to the linearities at the ends of the depletion region. To characterize the overall displacement in the V-I characteristics, we average the sensitivities of different normalized photocurrent point locations as the sensing capability of the LAPS. The average positive sensitivity and the average positive linearity are 45.83 mV/pH and 98.5%, respectively. The average negative sensitivity and the average negative linearity are 49.9 mV/pH and 98.64%, respectively. The difference in the V-I characteristics measured by positive travel and negative travel is caused by scan-direction-dependent hysteresis. Therefore, by combining the measurement results of positive travel and negative travel, we can obtain the average sensitivity and average linearity of the traditional VICM method. The average sensitivity and the average linearity are 47.87 mV/pH and 98.57%, respectively. Compared to using the point where the normalized photocurrent is 0.5 as the working point, sensitivity and linearity are reduced by 2.39% and 0.58%, respectively. Although the average sensitivity and the average linearity obtained by the VICM method are reduced, this means of characterizing the sensing ability of LAPS better reflects the change in shape and the overall displacement of the depletion region of the V-I characteristics during actual measurement. The entire calculation process involves data sampling and processing. It takes 3.2 s to sample 500 data points of the entire V-I characteristics. The data processing involves finding the maximum value, point-by-point normalization, and finding the working point, which takes approximately 0.15 s. Therefore, the time for a single V-I characteristics measurement is 3.35 s.

### 3.2. Gaussian Function Fitting Measurement Method

This section investigates and optimizes the GFFM method from six aspects: interference from the light source, the range of the bias voltage, the number of measurement points, the step value of the bias voltage, the type of the light excitation waveform and ion selectivity. Finally, LAPS using the GFFM method is used to measure sewage samples.

#### 3.2.1. Applicability

The bias voltage in this section uses the periodic inverted triangle from Section 2.4. The value range is set from −1.69 V to −0.334 V, and a single cycle contains 120 measurement points in steps of 11.3 mV. The illumination configuration is also used as described in Section 2.4. Measurement is performed on pH 8 buffer, and the single-cycle photocurrent curve is shown in Figure S4. As demonstrated in Figure S4, the original photocurrent data is fitted with a Gaussian function using the least squares method, achieving a fitting accuracy of up to 99% [26]. At the same time, other pH buffers are measured and fitted with a Gaussian function with a fitting accuracy of 99%. Instead of normalizing the V-I characteristics, the Gaussian function model accurately and specifically describes the depletion region of the V-I characteristics during actual measurement. The fitted curves are shown in Figure 3e. The results show that the width of the fitted curves gradually increased with the increase in the pH of the buffer, which is consistent with the theoretical expectation of Section 2.5. The fitted equations for each pH condition are as follows:

(4) $$\begin{array}{l} y_{\mathrm{pH}4}=0.8283\cdot e^{\frac{-{\left(x-115.7456\right)}^{2}}{119.0821^{2}}}\\ y_{\mathrm{pH}5}=0.8749\cdot e^{\frac{-{\left(x-119.7781\right)}^{2}}{123.8255^{2}}}\\ y_{\mathrm{pH}6}=0.9188\cdot e^{\frac{-{\left(x-119.7496\right)}^{2}}{128.5623^{2}}}\\ y_{\mathrm{pH}7}=0.9359\cdot e^{\frac{-{\left(x-123.7453\right)}^{2}}{133.3064^{2}}}\\ y_{\mathrm{pH}8}=0.9341\cdot e^{\frac{-{\left(x-124.7605\right)}^{2}}{137.1713^{2}}} \end{array}$$

According to Equation (4), we can extract c of each fitted equation and calculate the corresponding FWHM. The slope and linearity of the GFFM method can be obtained by linearly fitting the relationship between FWHM and pH, and the results are shown in Figure 3f. Sensitivity is obtained by multiplying the slope by the step value of bias voltage. Ten measurements are conducted under each pH condition. The experimental results show that the sensitivity is 85.65 mV/pH and linearity is 99.7%, as obtained by the GFFM method. The bias voltage scan of the GFFM method includes both positive travel and negative travel, so its sensitivity exceeds that of the traditional VICM method using the point where the normalized photocurrent is 0.5 as the working point. To compare with the GFFM method, we take the sum of the average positive sensitivity and average negative sensitivity as the sensitivity of the traditional VICM method and the mean of the average positive linearity and average negative linearity as the linearity of the traditional VICM method. So, the sensitivity and the linearity of the traditional VICM method are 95.73 mV/pH and 98.57%, respectively, and the LOD of the GFFM method based on the LOD definition of IUPAC is 0.18 pH. It can be determined from Section 3.1 that in actual measurement, the horizontal displacement at the middle position of the depletion region is significantly greater than that far from the middle position of the depletion region. The GFFM method depicts the overall displacement of all measurement points in the depletion region and conducts a comprehensive statistic of the horizontal displacements of all points between the normalized photocurrent point locations of 0.2 and 0.9. Therefore, the GFFM method contains more horizontal displacement statistics far from the middle position of the depletion region, significantly reducing its sensitivity. Although the sensitivity of the GFFM method is reduced, the detection linearity improves by 1.13%, and the actual pH resolution is superior to that of the traditional VICM method. The GFFM method utilizes the Gaussian function to fit the depletion region of the V-I characteristics for both positive and negative travel with high accuracy and transforms the horizontal displacement of the traditional unilateral V-I characteristics into the spread degrees of the fitted curves, which compensates for measurement errors in calculating the displacement of some working points due to some measurement point distortions, instrument measurement noise and environmental noise [27]. The GFFM method requires only a small amount of data from the depletion region, reducing the amount of data by 52%. As a result, the data sampling time decreases to 1.53 s. The data processing process involves performing Levenberg–Marquardt nonlinear least squares fitting, extracting parameters, calculating FWHM, etc. In addition, the FPU of the MCU is utilized to accelerate matrix operations, and the data processing time is significantly reduced and is only 0.1 s due to the small amount of data. Therefore, the single measurement time is only 1.63 s, which doubles the measurement speed compared to the VICM method. To examine reproducibility of the GFFM method among different devices, the sensitivities and linearities of five independent LAPSs are measured under the same working condition as above, and the results are shown in Figure 3g. The data indicates that the relative standard deviation of the sensitivities of the five groups of LAPSs is 0.57%, and the average linearity is 99.7%. According to our previous work [28], the relative standard deviation of the sensitivities of LAPSs is 5.12% and the average linearity is 98.9% using the VICM method. So, the GFFM method shows high repeatability. In addition to reproducibility, the short-term stability is also an important index to evaluate performance of LAPSs. Sensitivities and linearities of an independent LAPS using the GFFM method are recorded regularly for a week, as shown in Figure 3h. The experimental results show that the sensitivity of the LAPS still maintains 99.77% of the initial sensitivity, and linearity is not decreased one week later. At the same time, the sensitivity of the LAPS using the VICM method one week later only maintains 91.5% of the initial sensitivity according to our previous work [28]. Therefore, the GFFM method has good short-term stability for pH detection compared to the VICM method. As mentioned above, the direct quantitative comparison between the traditional VICM method and the GFFM method is shown in Table 1. It can be clearly seen that the overall performance of the GFFM method is superior to that of the traditional VICM method, demonstrating excellent pH detection capabilities.

#### 3.2.2. Resistance to Light Excitation Fluctuations

**Figure 4:**
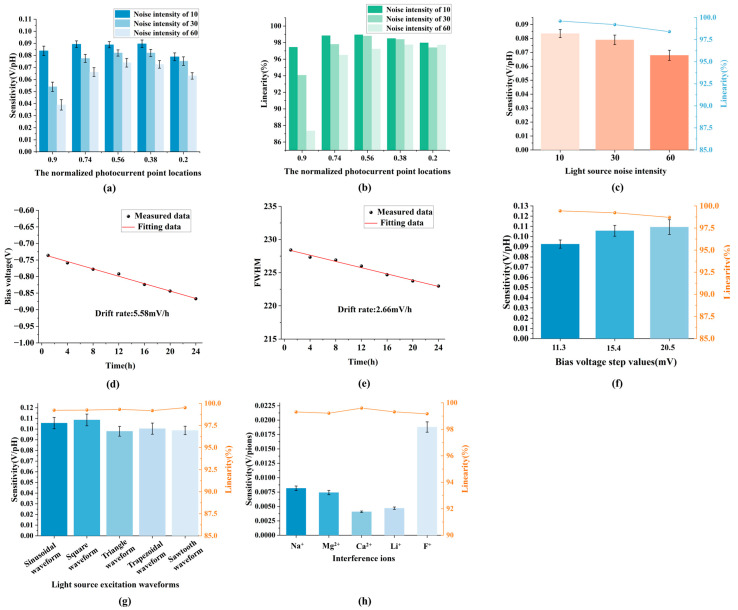
The anti-interference ability, optimization and ionic selectivity of the GFFM method. The detection sensitivities (**a**) and linearities (**b**) obtained by the traditional VICM method combining the measurement results of positive travel and negative travel under the illumination with noise. (**c**) pH detection results obtained by the GFFM method under illuminations with noise. (**d**) The drift property of the LAPS measured in a solution of pH 7 for 24 h using the traditional VICM method. (**e**) The drift property of the LAPS measured in a solution of pH 7 for 24 h using the GFFM method. (**f**) pH detection results of the GFFM method under different step value conditions. (**g**) pH detection results of the GFFM method under the illumination conditions of five typical excitation waveforms. (**h**) Interference ion detection results of the GFFM method.

Because the GFFM method gives statistical properties to the data, it can resist interference to a certain extent. Therefore, we designed experiments to compare the two methods for pH detection under the fluctuations in the light source. The drive circuit for the laser tube in this paper adopts the design from our previous work [29], which regulates the light power by controlling the current flowing through the laser. The drive current is regulated by the voltage output from a digital-to-analog converter (DAC) and the drive circuit. Therefore, we can introduce Gaussian white noise with intensities of 10, 30, and 60 into the DAC output voltage through microcontroller programming to artificially simulate light source fluctuations. DAC output voltages, i.e., the illumination voltages during one cycle, are shown in Figure S5. Under the illuminations at noise intensities of 10, 30 and 60 as described above, the detection sensitivities and linearities obtained by the traditional VICM method combining the measurement results of positive travel and negative travel are shown in Figure 4a,b. The corresponding sensitivities are 86.08 ± 3.15 mV/pH, 74.12 ± 3.42 mV/pH and 62.9 ± 3.77 mV/pH, respectively, which are 10.08%, 22.57%, and 34.29% lower than the initial sensitivity without noise. Similarly, the relative standard deviations (RSDs) are 3.65%, 4.61% and 5.99%, respectively, and the corresponding linearities are 98.36%, 97.3% and 95.32%, respectively, which are 0.21%, 1.29%, and 3.29% lower than the initial linearity without noise. The pH detection results obtained using the GFFM method under the illuminations with noise intensity of 10, 30, and 60 are shown in Figure 4c. The results show that the corresponding sensitivities are 83.51 ± 2.91 mV/pH, 78.98 ± 3.44 mV/pH and 67.91 ± 3.65 mV/pH, respectively, which are 2.49%, 7.78% and 20.71% lower than the initial sensitivity without noise. Similarly, the RSDs are 3.48%, 4.35% and 5.37%, respectively. The corresponding linearities are 99.6%, 99.2% and 98.4%, respectively, which decreased by 0.1%, 0.5% and 1.3% compared with the initial linearity without noise. Under illumination with noise conditions, the sensitivity degradation rates, linearity degradation rates and the RSDs of the GFFM method are significantly lower than those of the traditional VICM method, indicating that the GFFM method is resistant to interference from fluctuations in light source. In the case of low-noise illumination, the sensitivity of the GFFM method is close to the sensitivity of VICM method, the linearity of the former is improved by 1.24% and the RSD is decreased by 0.17%. When the noise intensity of the illumination increases to 30, the sensitivity and linearity of the GFFM method become significantly greater than those of the traditional VICM method, with an increase of 6.15% in sensitivity and 1.92% in linearity, and the RSD is decreased by 0.26%, which indicates that under the illumination of further increasing noise, the GFFM method performs better against noise interference. When the noise intensity of the illumination increases to 60, the sensitivity and linearity of the GFFM method are still substantially better than those of the traditional VICM method, with an increase of 7.37% in sensitivity and 3.13% in linearity, and the RSD is decreased by 0.62%, which shows that under the high-noise illumination condition, the sensitivity degradation ratios, the linearity degradation ratios and the RSDs of the GFFM method are greatly reduced, and this method can maintain high detection performance. However, real light source fluctuations may exhibit non-Gaussian patterns or long-term thermal drift. An experiment to verify the real light source fluctuation is conducted by continuously monitoring the drift rate of an LAPS subjected to a solution of pH 7 for 24 h. The results are shown in Figure 4d,e. The results show that the traditional VICM method exhibits a baseline drift of approximately 5.58 mV/h at the working point, while the FWHM of the GFFM method only drifts by 2.66 mV/h. Therefore, in the presence of actual external environmental disturbances such as fluctuating ambient light or thermal instability of the excitation source, the GFFM method exhibits greater resistance to interference.

#### 3.2.3. Optimization of the Bias Voltage Range, the Number of Measurement Points and Step Value

To further improve the measurement efficiency, we divide the complete depletion region into two equal parts based on the difference in charge carrier dynamics of the LAPS under reverse bias voltage, and the partitioning of the depletion region is shown schematically in Figure S6. The first half of the depletion region is designated as depletion I, and the second half of the depletion region is designated as depletion II. Since the displacement current in depletion I is greater than that in depletion II, the photocurrent in depletion II is lower, causing the V-I characteristic to exhibit a non-symmetric concave triangular shape, where the Gaussian model no longer captures the curvature sufficiently (fit residual increases and R^2^ drops). Therefore, we exclude “depletion II” from the fitting window and focus on “depletion I” to preserve robustness and accuracy. Based on the experiments in Section 3.2.1, the bias voltage range of depletion I in this section is set from −1.69 V to −1.012 V, and the number of measurement points in the bias voltage range is set to 60 to keep the step value of the bias voltage at 11.3 mV. At the same time, we further reduce the number of measurement points by increasing the step value of the bias voltage, i.e., controlling the range of the bias voltage to remain unchanged and increasing the step value of the bias voltage from 11.3 mV to 15.4 mV and 20.5 mV. pH detection results of the GFFM method at bias voltage step values of 11.3 mV, 15.4 mV and 20.5 mV are shown in Figure 4d. When the step value is 11.3 mV, the sensitivity and the linearity of the LAPS are 92.58 mV/pH and 99.44%, respectively. When the step value is set to 15.4 mV, the sensitivity and the linearity of the LAPS are 105.64 mV/pH and 99.23%, respectively. When the step value increases to 20.5 mV, the sensitivity and the linearity of the LAPS are 109.26 mV/pH and 98.71%, respectively. This indicates that the GFFM method maintains high detection accuracy under the conditions of concentrating the detection area in the depletion I region and reducing the measurement data by 93%. There are two reasons for the improvement in sensitivity: first, depletion II has more Gaussian characteristics than depletion I after partitioning; second, discarding depletion II and the reduction in measurement points reduces the horizontal displacement statistics at the middle position far from the depletion region, so the sensitivity of the GFFM method increases with the increase in the bias voltage step value and the decrease in the number of measurement points, and it exceeds the sensitivity of the traditional VICM method. It is worth noting that the detection linearity of the GFFM method decreases slightly when the step value of the bias voltage increased to 20.5 mV, but it stays above 98%.

#### 3.2.4. Optimization of Light Excitation Waveforms

To optimize the excitation waveforms of the light source for the GFFM method, we explore the effects of five typical excitation waveforms, such as a sinusoidal waveform, square waveform, triangle waveform, trapezoidal waveform and sawtooth waveform on the pH measurement results of the LAPS. Except for excitation waveforms, the light power, wavelength, light spot size, and modulation frequency of the light source are the same [29]. Based on Section 3.2.3, the sensitivity obtained from the GFFM method at a step value of 20.5 mV is greater than that at a step value of 15.4 mV, but the linearity is reduced. Therefore, we set the bias voltage to be in depletion I with a value ranging from −1.69 V to −1.012 V in a step of 15.4 mV to optimize the light excitation waveforms. Figure 4e shows the pH measurement results obtained by the GFFM method when a sinusoidal waveform, square waveform, triangle waveform, trapezoidal waveform and sawtooth waveform are used as the excitation waveforms of the light source, respectively. The linearity is 99.23% and the sensitivity is 105.64 mV/pH when the excitation waveform is sinusoidal. When the waveform excited by the light source is triangular, the linearity is 99.33% and the sensitivity is 97.94 mV/pH. When the waveform excited by the light source is square, the linearity is 99.25% and the sensitivity is 108.57 mV/pH. The linearity is 99.19% and the sensitivity is 100.4 mV/pH when the waveform excited by the light source is trapezoidal. The linearity is 99.52% and the sensitivity is 98.71 mV/pH when the waveform excited by the light source is sawtooth. The results show that LAPSs using the GFFM method exhibit a high sensitivity of more than 97 mV/pH, a linearity of more than 99% under the conditions of short bias voltage range, a small number of detection points, a large step value of the bias voltage and five typical excitation waveforms.

#### 3.2.5. Ion Selectivity

The sensing film of the LAPS in this paper is Si_3_N_4_ film. Therefore, investigating the response mechanism of the LAPS to pH and its selectivity to interfering ions means investigating the recognition mechanism of Si_3_N_4_ film. The pH-selective recognition mechanism of Si_3_N_4_ sensing film originates from its amphoteric surface chemistry. Upon hydration, the Si_3_N_4_ surface undergoes hydrolysis, forming a silanol (Si-OH) and amine (Si-NH_2_) terminated layer. These functional groups exhibit protonation/deprotonation reactions in response to hydrogen ion activity in the adjacent solution. In acidic environments, Si-NH_2_ accepts protons to form positively charged ammonium species (Si-NH_3_^+^), while under alkaline conditions, Si-OH dissociates to generate negatively charged silanolate moieties (Si-O^−^). This pH-dependent ionization alters the surface charge density at the solid–liquid interface, establishing an electrochemical potential across the film. According to the site-binding model, this potential shift follows the Nernstian relationship. In this section, we introduce Na^+^, Mg^2+^, Ca^2+^, Li^+^ and F^−^ as interfering ions to detect the sensing performance of the LAPS using the GFFM method. The bias voltage range is set from −1.69 V to −1.012 V, the step value of the bias voltage is 15.4 mV and the light source excitation waveform is square. The test solutions are prepared using CaCl_2_ solution, NaCl solution, MgCl_2_ solution, LiCl solution, NaF solution and deionized water, respectively, and sensitivities and linearities are summarized in Figure 4f. The sensitivity of the LAPS to the impurity ions such as Na^+^, Mg^2+^, Ca^2+^ and Li^+^ is 8.16 mV/pNa, 7.42 mV/pMg, 4.08 mV/pCa and 4.7 mV/pLi respectively, all of which are significantly lower than the detection sensitivity for H^+^. This confirms that the LAPS with a Si_3_N_4_ sensing film exhibits exceptional pH selectivity with strong rejection of Na^+^, Mg^2+^, Ca^2+^ and Li^+^ interference. However, the sensitivity of the LAPS to fluoride ions reaches 18.8 mV/pF, and the interference in hydrogen ion detection significantly increases. Because F^−^ forms strong hydrogen bonds with the surface Si-OH, which interferes with pH detection, fluorine-containing samples should avoid using Si_3_N_4_-LAPS to measure pH. It is recommended to use other sensing films instead. The selectivity over interfering ions arises from the Helmholtz layer’s preferential interaction with H^+^/OH^−^ ions, attributed to the high hydration energy of small ions and steric hindrance of non-H^+^ ions near the binding sites. Furthermore, the covalent bonding and thermodynamic stability of Si_3_N_4_ minimize ion diffusion into the bulk film, ensuring potentiometric responses exclusively governed by pH variation.

#### 3.2.6. Sewage Sample Measurement

**Figure 5:**
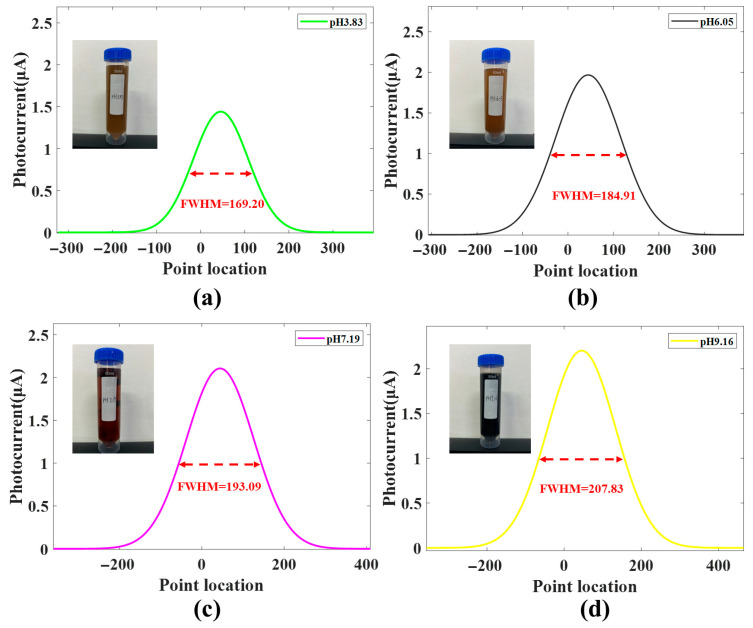
The sewage sample measurement results using the GFFM method. The measurement results of the pH values of four sewage samples with pH calibration values of 3.83 (**a**), 6.05 (**b**), 7.19 (**c**) and 9.16 (**d**) using LAPS based on the GFFM method. The inset is a photograph of the sewage sample.

To test the pH detection performance of the GFFM method in real-world scenarios, this paper applies an LAPS based on the GFFM method to detect sewage samples. A sewage sample with a pH of 6.05 calibrated using a standard pH meter is used as the reference sample. The bias voltage and light source configuration are the same as in Section 3.2.5. The V-I characteristic of sample is tested and fitted using Gaussian function model, and the fitted curve is shown in Figure 5. The test samples are sewage samples with pH values of 3.83, 7.19 and 9.16, calibrated using the same standard pH meter. The GFFM method is used to test these three samples, and the fitted curves are shown in Figure 5a,c,d. The results show that the FWHM values for the four samples are 169.20, 184.91, 193.09, and 207.83, respectively. The FWHM differences between pH 3.83, pH 7.19, pH 9.16, and the reference sample are −15.71, 8.18, and 22.92, respectively. Multiplying the FWHM differences by the bias voltage step value converts the FWHM differences into the voltage differences, with values of −241.934 mV, 125.972 mV and 352.968 mV, respectively. Dividing these values by the sensitivity of 108.57 mV/pH yields the corresponding pH differences from pH 6.05. Therefore, the LAPS measures the pH values of the three sewage samples with pH calibration values of 3.83, 7.19 and 9.16 to be 3.82, 7.21, and 9.30, respectively. The errors in measuring the pH values of sewage samples with the LAPS are only 0.26%, 0.27% and 1.7%, respectively. The reasons for the increased detection error under high pH conditions may be as follows: (1) the sewage samples’ matrix effects such as variable ionic strength, multicomponent buffering, and organics can alter the local interfacial environment and cause measurement errors; (2) at highly alkaline pH, the strong deprotonation of the surface sites causes the upper part curve of the depletion I during detection to approach a saturated state using the GFFM method. To address the increase in measurement errors caused by an increase in pH and non-ideal curve shapes, we plan to develop a mixed Gaussian–Lorentzian fitting method in the next step to expand the applicability of the GFFM method under extreme conditions. Importantly, the reported 1.7% error at pH 9.16 corresponds to a small absolute pH error and remains within practical accuracy for many field applications. In practical applications, LAPS based on the GFFM method can measure pH values with high accuracy and speed. Through further research, it can be used for high-precision, rapid detection of other biochemical species related to pH.

## 4. Conclusions

This paper presents a new pH measurement method applied to an LAPS. The method makes the LAPS operate in the depletion region by applying a periodic inverted-triangle bias voltage and forms a nearly symmetric Gaussian-like V-I characteristic, which can be fitted using a Gaussian function to obtain the fitting equation. Based on the fitting equation, we can calculate FWHM for each pH measurement condition and obtain a linear relationship between FWHM and pH, thus obtaining the sensitivity and linearity characteristics of the pH measurement. Compared with the traditional VICM method, the GFFM method can reduce the impact of measurement point distortion and noise fluctuations on measurement performance. The method requires only a small amount of photocurrent data in the depletion region to realize pH measurements with high sensitivity, high linearity, strong specificity, strong immunity to interference, and universal waveform excitation. This method not only extends the model of LAPS for pH measurement but also provides the theoretical foundation and the experimental basis for high-precision, rapid pH detection and other biochemical species detection marked by pH.

## Figures and Tables

**Table 1:** The performance comparison of LAPS using the VICM method and the GFFM method.

	The VICM Method	The GFFM Method
Sensitivity (mV/pH)	49.04	85.65
Linearity (%)	99.15	99.7
LOD (pH)	0.32	0.18
Single measurement time (s)	3.35	1.63
Reproducibility (%)	5.12	0.57
Short-term stability (%)	91.5	99.77

## Data Availability

Data are contained within the article and Supplementary Materials.

## Supplementary Materials

The following supporting information can be downloaded at: https://www.mdpi.com/article/10.3390/s26051465/s1, Figure S1: Fabrication of LAPS chip. Figure S2: The microscopic morphology and EDS surface scan images of the cross-section of LAPS chip at 2000× (a,b) and 100,000× (c,d) magnification. Figure S3: Packaging of LAPS chip. Figure S4: Single-cycle photocurrent and the Gaussian function fitting curve of LAPS by measuring pH 8 buffer. Figure S5: The illumination voltage at noise intensities of 10, 30 and 60 during one cycle. Figure S6: Schematic diagram of the partitioning of the depletion region.
