# Supplementary material for: Gaussian-Fitting-Enabled High-Accuracy pH Detection for Light-Addressable Potentiometric Sensor
Source: Sensors (Basel). 2026 Feb 26;26(5):1465. doi: 10.3390/s26051465 (PMC12987349; doi:10.3390/s26051465)
Supplement: Supplementary file 1 [file sensors-26-01465-s001.zip › sensors-4135130-supplementary.pdf]

Supplementary Material

Gaussian-Fitting-Enabled High-Accuracy pH Detection for Light-Addressable Potentiometric Sensor

Jie Tan [1,*], Zigeng Huang [1], Bin Sun [1], Xin Cao [1], Zijie Tang [1], Guomao Yan [1], Jiangze Ren [1], Shibin Liu [2], Yinghao Chen [2], Guifang Li [2], Xueliang Li [3] and Dong Chen [4]

[1] *School of Electrical Engineering and Electronic Information, Xihua University, Chengdu 610097, China; zghsczt66@163.com (Z.H.); 13721360102@163.com (B.S.); cuitcx@163.com (X.C.); 18180318380@163.com (Z.T.); yanguomao11@163.com (G.Y.); m18398422628@163.com (J.R.)*

[2] *School of Electronics and Information, Northwestern Polytechnical University, Xi'an 710072, China; liushibin@nwpu.edu.cn (S.L.); 18829589303@163.com (Y.C.); gfli@nwpu.edu.cn (G.L.)*

[3] *School of Mechanical and Electrical Engineering, Zhoukou Normal University, Zhoukou 466001, China; zhf870721@zknu.edu.cn*

[4] *School of Electronics Engineering, Xi'an University of Posts and Telecommunications, Xi'an 710121, China; dchen402@126.com*

* Corresponding author.

E-mail address: tanjie_xihua@163.com

Process S1:

(a). The silicon wafer is first cleaned in a standardized process and then blown dry under nitrogen;

(b). The silicon wafer is placed in a high-temperature oxidation diffusion oven, and a mixed gas of nitrogen, low flow oxygen, and water is introduced. The temperature is raised to 1050 °C, and the flow rate of the mixed gas is increased to continuously react with trichloroethylene. Then the mixed gas is switched to nitrogen gas, and annealing treatment is performed. Finally, the diffusion oven cools down to room temperature and the silicon wafer is pulled out of the diffusion oven;

(c). Subsequently, low-pressure chemical vapor deposition (LPCVD) is used to deposit a 50 nm Si_3N_4 thin film as a sensing film on the silicon dioxide insulator layer of silicon-based LAPS. The reaction is carried out in a 4374 low-pressure chemical vapor deposition oven at a temperature of 800 °C, using silane gas and ammonia gas as reaction gases. Finally, the deposition oven cools down to room temperature and the silicon wafer is pulled out of the deposition oven;

(d). Immerse the semiconductor substrate with a silicon nitride oxide film in anhydrous ethanol, and use an ultrasonic cleaner for vibrational cleaning for 5 minutes. After cleaning, rinse the substrate with deionized water and finally dry it with nitrogen gas. Set the speed of the spin coater to 2500 rpm and spin the photoresist coating on the silicon wafer surface. After spinning, place the silicon wafer on an hot plate and bake it for 3 minutes at a temperature of 105°C. At this point, a single-sided spin-coated substrate is obtained to protect the silicon nitride oxide film from being corroded by subsequent processes;

(e). The single-sided photolithography substrate obtained in step (d) is floated on a 49% hydrofluoric acid solution for etching. To ensure that the exposed silicon nitride and silicon dioxide are completely removed, the etching time should be greater than 4 minutes;

(f). The sample are cleaned with acetone and anhydrous ethanol to remove the photoresist. Set the speed of the spin coater to 4000 rpm and spin the photoresist on the etched surface of the silicon wafer. After spinning, place the silicon wafer on a hot plate surface to heat and cure the photoresist. The baking time is 3 minutes, and the hot plate is set to 105°C. At this point, the silicon wafer is coated with photoresist on both sides, and the thickness of the two layers of photoresist is not the same;

(g). Open the lithography machine, place the silicon wafer inside for back exposure, and set the exposure time to 29 seconds. After exposure, develop the semiconductor substrate using a 0.8% potassium oxide solution for 2 minutes. After development, place the substrate on a hot plate at 105°C and bake for 3 minutes.

(h). For working electrodes, Cr and Au are sequentially deposited on the back side of the silicon wafer by DC and RF magnetron sputtering. The working vacuum of magnetron sputtering is 0.9 Pa, the working temperature is room temperature, the power and the argon flow of DC magnetron sputtering are 86 W and 15 sccm, respectively. The power and the argon flow of RF magnetron sputtering are 120 W and 40 sccm, respectively;

(i). After magnetron sputtering is completed, soak the sensor substrate in acetone for 5 minutes to dissolve the photoresist on the back side of the substrate. Then place the substrate in anhydrous ethanol and clean it with an ultrasonic cleaner for 5 minutes. At this point, turn the vibration power knob on the ultrasonic cleaner to the minimum setting to avoid damage to the semiconductor substrate caused by excessive vibration power. After cleaning, use nitrogen to blow the substrate clean.

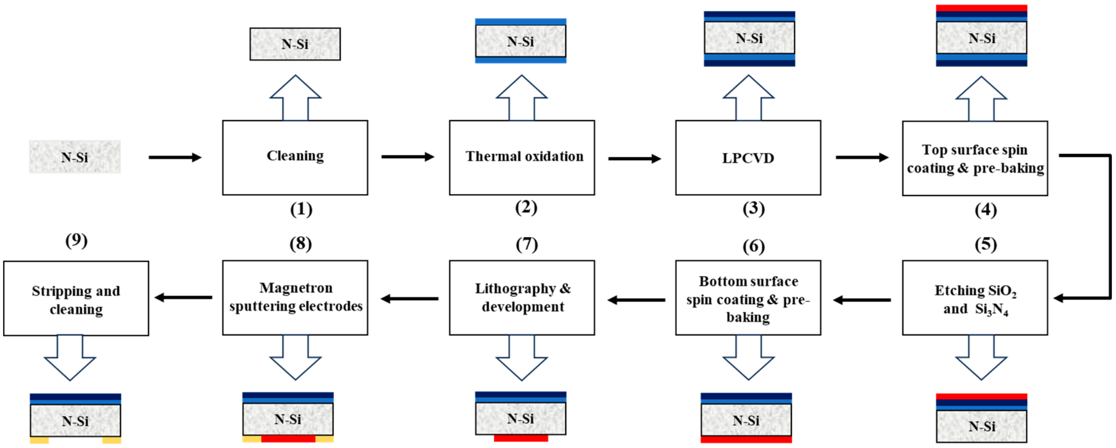

Figure S1. Fabrication of LAPS chip.

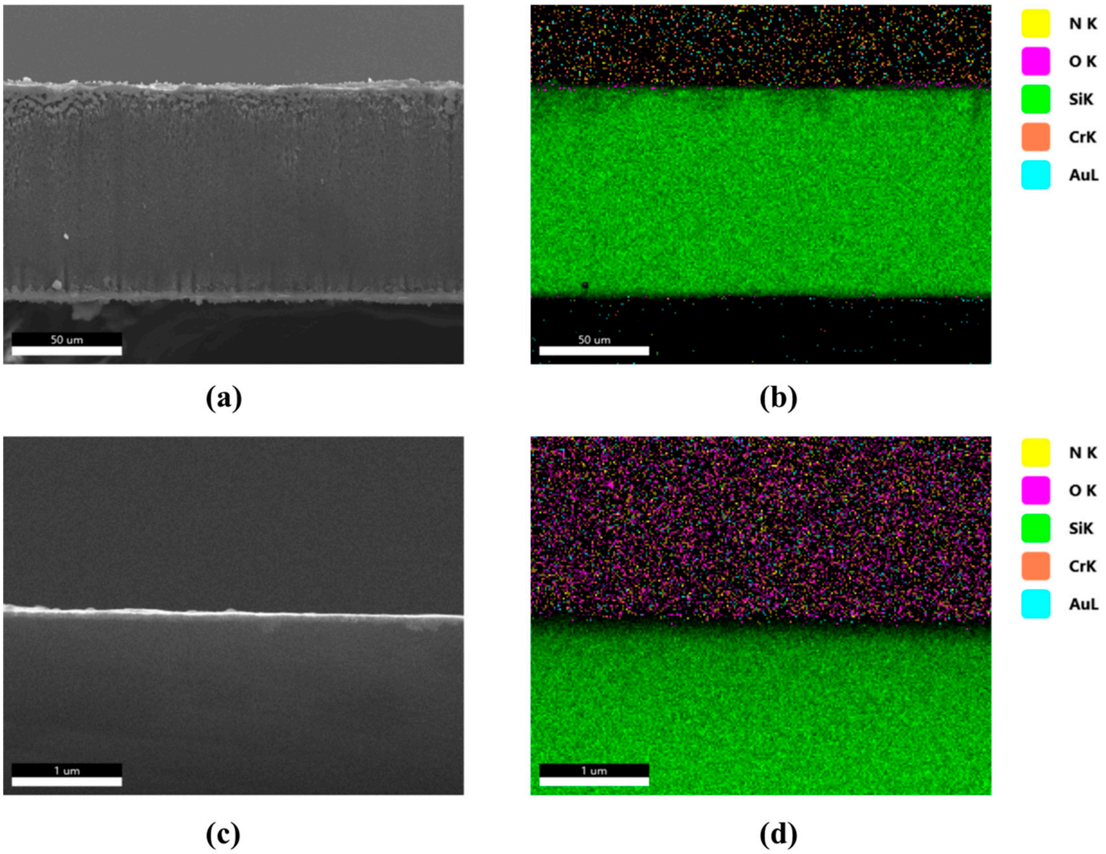

Figure S2. The microscopic morphology and EDS surface scan images of the cross-section of LAPS chip at 2,000x (a-b) and 100,000x (c-d) magnification.

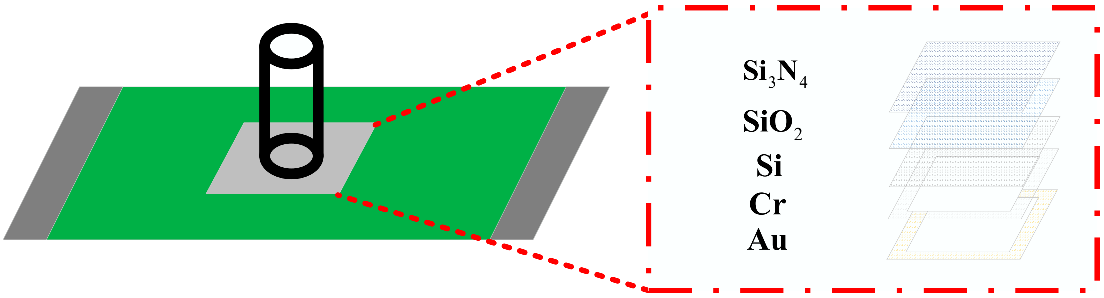

Figure S3. Packaging of LAPS chip.

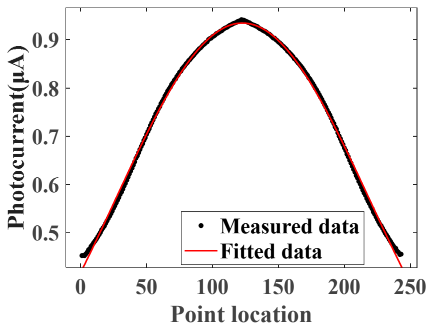

Figure S4. Single-cycle photocurrent and the Gaussian function fitting curve of

LAPS by measuring pH 8 buffer.

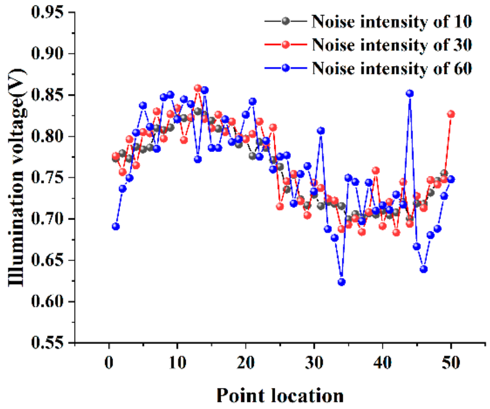

Figure S5. The illumination voltage at noise intensities of 10, 30 and 60 during one cycle.

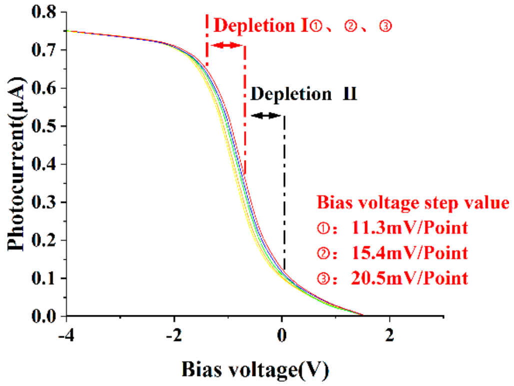

Figure S6. The schematic diagram of the partitioning of the depletion region.
